# Safety and Occupational Health: Challenges and Opportunities in Emerging Economies

**DOI:** 10.4103/0019-5278.40808

**Published:** 2008-04

**Authors:** Hital R Meswani

**Affiliations:** Executive Director, Reliance Industries Limited, Maker Chambers IV, 222, Nariman Point, Mumbai 400 021

## Ladies and gentlemen

A very warm good evening to all of you and thank you Dr.Ramnik Parekh for a very generous introduction.

It is my privilege to share the dais with such eminent professionals from the Occupational health fraternity at the 58th National Conference of the Indian Association of Occupational Health.

I have gone through the agenda of the conference and it is indeed rich in content. I am sure that in the next 3 days you will be treated to a feast of knowledge from the many experts who have gathered here from India and abroad to exchange ideas and share their views on a subject that is so critical to all nations and more so to emerging economies.

I compliment the organisers for scheduling this conference at such an appropriate time when emerging economies or more popularly known as BRIC countries (Brazil, Russia, India, China) are fuelling the global economic growth engine and when every Fortune 500 company is focusing its future investments in these emerging markets.

Safety and Occupational health is of paramount importance to all emerging economies at this juncture and the presence of such a large number of professionals here at this conference only lends credence to this fact.

In line with the theme of the conference, over the next 20 minutes, I will try to address the issues, challenges and strategies for enhancing the efficacy of Occupational Health & Safety aspects in emerging economies.

I will also give you a glimpse of our experience at Reliance in putting the strategies for success into action.

## 2.0 OHS?

### 2.1 History of OHS

From its roots in Europe, as early as 16^th^ century the concept of Occupational Health has come a long way only to become a very integral part of the world economy & society.

In 1906 in Milan an important step for the modern global workforce was the formation of the Permanent Commission on Occupational Health later renamed the International Commission on Occupational Health (ICOH). Today, ICOH is the world's leading international institution in the field of occupational health with a membership of 2,000 professionals representing nearly 100 countries.

Occupational health & safety is not a new concept for India. The existence of the Indian Association of Occupational Health (IAOH) for the past 60 years since 1948, is a testimony of the same.

### 2.2 WHY OH?

We are all aware of the several benefits of OH&S: increased productivity, higher quality of work, increased workforce morale and reduced employee turnover, just to mention a few.

You have heard of “Maslow's hierarchy of needs”, that human beings first look to satisfy their physiological and safety needs before aiming for social, esteem and growth needs.

In line with this an individual at a workplace, also looks for health and safety first and its absence can be a serious de-motivator.

Let me share with you some facts from the International Labour Organization’s report on the global burden of occupational health illnesses:

1. Of the world's 2.7 billion workers about 2 million deaths are attributable to occupational diseases and injuries
2. About 4% of the GDP is lost due to occupational diseases and injuries.

Health, Safety and Environment are enablers for economic development. All the three aspects of business are measured by their negative impact on people, assets and environment.

The damage is acute in the area of safety, chronic in the area of occupational health and persistent in the case of environment.

While the impact of safety is felt immediately, the effects of OH related diseases are felt over generations. A prime example in India is the Bhopal gas tragedy.

### 2.3 Safety in Conjunction with OH

Occupational health cannot be addressed in isolation but rather has an important relationship with Safety.

The various aspects of safety management actually aim at mitigating the risks to an individual at the work place through various methods like hazards analysis, Risk Assessment and safety information.

For example at Reliance every small change to the manufacturing plant process undergoes a series of evaluation with respect to hazard analysis and ergonomics. This gives us immense confidence in the robustness and high level of safety of our processes & assets.

I firmly believe that the best way to prevent accidents and occupational health hazards is at the design stage itself by incorporating best practices and configuring work processes.

People cannot react to the damage that they cannot see immediately, let me give you an example -

Boiling water frog: If you heat a frog in a beaker of water, gradually it will die because it becomes complacent, but if you throw a frog into a beaker of boiling water it will jump off immediately.

This analogy can be extended to OH and safety. For all accidents we see we tend to put in mitigation efforts. But for OH illnesses which have a long gestation time we tend to become like the frog in a gradually heated beaker.

So it is important to acknowledge that safety and occupational health always work in conjunction.

While OHS is important for all nations, emerging economies have certain peculiarities which make the implementation of OHS initiatives a complex issue. India and China together account for nearly a third of the world's population.

## 3.0 IMPORTANCE OF OHS TO EMERGING ECONOMIES

Today emerging economies like China and India have caught the attention of the developed world not only for the vast potential the markets here hold for investment but also for the huge mass of low cost labour.

There is a huge flow of Foreign Direct Investment in emerging economies leading to the inception of huge infrastructure projects. Also many multinationals are setting up a manufacturing or service base to cut down on manpower cost.

The magnitude and complexity of the workforce required to drive the economic growth makes it imperative for emerging economies to focus on occupational health to sustain the economic growth.

### 3.1 Characteristics of Emerging Economies

Emerging economies have certain unique characteristics which differentiate them from the developed world. These unique characteristics are:

1. High growth rates
2. Large population density
3. Low cost low knowledge workers
4. Poor literacy rates
5. About 70% of economically active population works in agriculture sector
6. Employment in the unorganized sector may reach up to 70% with contribution to the GDP ranging between 10 to 60%

These economies bear more than 80% of the global burden of occupational disease and injury.

According to a report by the International Labour Organisation (ILO), in the year 2001, 37% of the total fatal occupational injuries are contributed by China and India alone as compared to the 5% by developed market economies.

As regards Lost time injuries, 36% are contributed by China and India alone as compared to the 4.5% by developed market economies.

Historically it has been seen that infrastructure spending determines the sustained economic growth of any nation. Many countries have found their growth rates stagnating for the want of good infrastructure.

The emerging economies need to realize that Occupational health is a similar strategic enabler to their economic growth. Without sufficient focus and spending on occupational health emerging economies too would find their growth stagnating.

I am reminded of a term frequently used in management parlance, called BPR or Business Process Re-engineering, which refers to a change for the better in running a business.

Few companies think of BPR when the going is fine. Most of them attempt this as a salvage measure when they run into trouble, by which time they neither have the time nor the energy or resources to manage any transition.

OH&S in emerging economies is in a similar phase. They need to apply BPR right now when the going is good.

The emerging economies need to take a quantum jump to be at par with the developed world. But for this to happen various challenges need to be overcome and diverse issues need to be addressed in an effective manner.

Let me first summarize these issues and then elaborate on them.

### 3.2 The key issues are

1. Leadership mindset and corporate commitment
2. Life cycle approach to OH, investment in infrastructure and management systems
3. Awareness, Education and Training
4. Regulatory framework and compliance
5. Use of Technology as a strategic enabler

#### 3.2.1 Leadership Mindset and Corporate Commitment

In emerging economies there is little convergence of OHS with business

Business goals are mostly aligned with productivity and increased profits.

Most organizations fail to recognize OH as a strategic enabler of sustained economic growth.

Also business performance is not measured with respect to OHS. As a result there is no incentive for stakeholders to improve on the OHS performance.

Many organizations also view investing in OHS as a luxury or one of those things that are required for compliance.

Certain industries also view OHS initiatives to be at cross-purposes with productivity and profit enhancement. What these industries miss out is that a safe work environment would in fact go a long way to increasing the productivity of their employees and in turn that of the company.

Also companies fail to recognize a good OHS record can not only be a strategic differentiator but also a source of competitive advantage.

Poor quality of assets is another issue. Most of the assets are out-dated and are not equipped to address the ergonomic demands of OH. Also many assets are made to ‘sweat' beyond their capability and result in increased probability of OH injuries.

For many organizations an annual medical check-up is considered as the ‘Be all' and ‘End all' of all OHS.

Friends, commitment to the OHS initiative has to start from the top. Till the top management does not invest time and resources to OHS, no OHS initiative can be successful.

The top management has to be convinced of the economic benefits of OHS initiatives and convert their “chalta hai” attitude to that of a “ can do”.

The management needs to state its objectives clearly and demonstrate leadership commitment to the entire organization.

Further organizations should make OHS an integrated part of performance measurement.

It is no secret that what cannot be measured cannot be improved.

The OH&S management program should include proactive measures like near miss reporting, accident investigations, risk assessment, auditing for compliance, emergency management programs, mock drills, benchmarking with leading companies in the field of OH&S, using inherently safe technologies, training and reliability programs.

All this should translate into investing in technology, infrastructure, education and training.

All organizations should strive to have “Build a Safe Beginning” which means starting with health and safety as a core value well before the first day of work.

By core value, I mean that health and safety consciousness must be an essential value, not just a priority, so that it is constantly thought of and actively used.

I emphasize this point because when safety – or any workplace activity – is a “priority”, it can be shifted.

For instance, we've all seen the slogans about “Health and safety being number one” and so on; however when workplace financial pressures get bad and there are cutbacks, or conversely, even when times are good and workers are overly busy, focus on safety and health may be short-changed.

However, if health and safety is a core value, then it is part of the mandatory elements of the functioning of the organization - an automatically assumed and ingrained business consideration that is not debated or compromised because it is truly at the core of workplace endeavours.

Safety and health then becomes more than merely “required” - it is, in fact, endemic to the enterprise as much as breathing is to a human. Safety as a core value is what we must all continue striving for, so at the end of the working day, all people come home safe.

#### 3.2.2 Life Cycle Approach to Oh, Infrastructure and Management Systems

Any new process goes through the cycle of conception-design-engineering-construction and commissioning.

In the life cycle approach to OH we should try to incorporate safety at every stage of the implementation of a process or product.

For example when evaluating technologies for the manufacture of a particular product, it should be ensured that inherent safe design is in place to address the use of hazardous chemicals in the process.

3D plant modelling allows for the best-in-class ergonomics to be incorporated in plant layout to prevent OH illnesses & injuries to the manufacturing plant personnel.

Infrastructure for OH is required on 2 fronts. 1) for the prevention of OH related injuries and 2) For provision of the most effective treatment in case injuries/illness do occur.

OH requires sufficient level of hardware in terms of diagnostic equipment, emission level detectors, sound level meters to identify sources of OH related diseases and also to carry out a proper diagnosis.

The more important infrastructure is that of trained manpower. Today the biggest challenge for OH to succeed in emerging economies is the availability of trained OH professionals.

Today India has a requirement of 8000 OH professionals. But at the present rate of graduations in OH this number can be achieved only in 3 decades.

Also of 240+ Medical council of India certified medical schools only a handful offer post-graduate courses on occupational health.

The concept of paramedics is less understood and there is an urgent need to develop this as a means to provide emergency medical response.

Also what is required is a management system for establishment of clear roles and responsibilities for implementation of OHS initiatives. The management system is also required to set up a program for OHS with clear goals and strategies to meet these goals.

#### 3.2.3 Awareness, Education and Training

The biggest worry for emerging economies is a huge working population with low literacy rates and an abysmal awareness of occupational health hazards.

To compound this problem is an attitude of acceptance of injuries and accidents as a part of life. This can be even seen on an everyday basis when people recklessly cross busy roads paying scant respect to traffic rules

There is also short a term focus on earning the daily bread which takes precedence over a safe work environment.

The need of the hour is to spread the awareness of occupational hazards to the large work population. They need to be sensitised to the long-term detrimental effects of the environment that they work in and what precautions can be taken to mitigate these effects.

During one of my visits to a manufacturing facility abroad I was taken through a mandatory awareness session of the hazards prevalent in that particular manufacturing site. This reveals the level of commitment in the developed world where even an outsider is made aware of the hazards. This level of awareness is sorely missing even in the employees of companies in the emerging economies.

But things cannot only stop at creating awareness. It has to be followed up with training.

Here I would like to cite a simple but relevant experience at our Jamnagar refinery. During the construction phase there was heavy movement of vehicles. Drivers were ignorant of the need to wear seat belts. It was through regular training sessions that the drivers were sensitised to the need of wearing safety belts.

This was no meagre achievement considering the literacy level of the drivers. Today if you visit the Jamnagar site, you are likely to be reminded by the driver to put your seat belt on before you can be driven to your destination.

People also need to be sensitised to the importance of Personal Protective Equipment as a means of first line of defence.

OHS awareness should be started at school level to ensure that the future working population is well aware of the occupational hazards and their rights to a safe work environment.

Integration of health and safety in all curriculums is essential and this task should be taken up on priority to ensure a safer working environment.

But to do all this we need a large trained force of OH professionals.

There is an urgent need to have more professional institutes that can impart the training to create a large pool of OHS professionals. This can be achieved through corporate and governmental initiatives.

#### 3.2.4 Regulatory Framework and Compliance

In emerging economies as compared to the developing countries, the existing regulatory and compliance mechanisms are not so stringent.

In many developing nations there are multiple authorities looking after OHS. There is no single authority to address the OHS requirements of the various industries.

There is no mechanism for ensuring reporting of incidents and as a result official statistics compare favourably with the OHS performance of developed countries.

There is no penalty for non-compliance and often, statutory procedures are such that there are more penalties for disclosure than for non-reporting.

Also regulations in many industries is not evolved, not detailed out and not complied with through audits, monitoring systems etc.

The policy makers too are driven largely by the need to address ‘more pressing' social and health issues that are politically less complicated and more saleable to the general public.

The need is therefore to have a single point responsibility with adequate authority to ensure compliance and also to disseminate OHS information to the industry.

This will also ensure capturing of all OHS data to understand developing trends in occupational diseases and thus implement measures to prevent recurrence.

I also strongly feel that a regulatory framework, though necessary, can only act as a catalyst to the process of OHS implementation. The main enabler will be the acceptance of all stakeholders to comply with the existing rules and regulations. Without this no amount of regulations can help implement OHS.

#### 3.2.5 Technology as a Strategic Enabler

Today we live in a technology enabled world. The power of technology can be harnessed to enable the effective implementation of many OHS initiatives

Today a host of document and MIS software exist that can churn out statistics with respect to OH disease, age wise statistics, management level wise statistics (upper management tends to have higher BMI and hypertension). All this can make studying patterns in OH extremely easy and fruitful.

The automation of hazardous processes, the development of smart detectors for emissions and radiation control devices can help in faster and accurate detection of potential sources of OH illnesses.

There are applications available which enable “virtual classrooms”i.e a trainer at one corner of the globe can train a group located in another corner.

Hypermedia offers new possibilities for visualizing work situations and workplaces as well as evaluating, planning, and developing work. For example, the user can control a situation just as if actually working. Some examples are cockpit simulators, training software for catastrophes, simulation of serious accidents, work tutorials and guides for industry, and operation manuals for machines.

Hypermedia can also help in providing information and instructions for workplace design. One application is an easy-to-use interface for some large database, for example chemical safety data sheets, legislation, and standards

The concept of OHS cockpit has now been introduced. It is akin to an ICU where all key emissions of hazardous substances are continuously monitored in a central location.

Friends, let me now take you through what we have experienced in our implementation of OHS at Reliance

## 4.0 The Reliance Experience

Reliance has various hazardous operations in the refining and petrochemical sector.

We have a Health Safety and Environment policy that guides all our activities in manufacturing.

Our commitment is “Safety of a person overrides all production targets.” We firmly believe all occupational illnesses and injuries are preventable.

Being a major producer of petroleum products and several petrochemicals and polymers, Reliance places high importance of OH&S in all its activities – right from conceptualisation of the process till the final product reaches to the consumer.

All plants are designed with adequate safeguards for process safety. Our 3D modelling and plant layout processes take into account the safety regulations with respect to location of equipments, storages etc..

Any process modifications done during the operating cycle of the plant undergo various rigorous safety, environment and health scrutiny before implementation.

We benchmark ourselves against the best practices and meet the requirements. We follow the strictest standards applicable globally.

We invite international agencies like Shell, Dupont and British Safety council for regular external audits.

Safety and health issues are reviewed daily at the top level meetings.

We also encourage people to spot and report unsafe behaviours and unsafe situations the risky jobs and reward them.

All our manufacturing units are OHSAS 18001 certified and various six sigma projects are also taken up in OH&S front.

100% medical compliance and personal protective equipment are now taken for granted.

The focus has shifted to process management and imbibing OH&S in the ethos.

Our OH&S professionals have implemented a simple yet innovative idea to identify and reduce occupational health hazards.

Every year each plant takes up a project on an Occupational Health issue that has a high impact.

The project could be on any of the occupational health issues: work place exposure to chemicals, heat stress, ergonomics, back injury prevention, high noise levels, obesity, etc.

We continue to be satisfied with the results of this initiative.

What started as a simple one project at one site has snowballed into a mass movement and several plants at all sites are doing it earnestly and reaping the benefits of improved quality of life at the workplace.

These OH&S projects have been named CASH – Change Agent for safety health.

The concept is so simple that any industry – big or small can implement it and get substantial benefits.

There is enthusiastic participation of all employees.

1200 CASH projects were implemented across all Reliance manufacturing locations in the year 2007.

Today Reliance is considered a front runner in the field of OHS and various awards and accolades have been bestowed on us by various prestigious international organizations. However we are not satisfied and we have a vision to be better than the best and take a quantum leap to widen the gap between us and our global competitors.

In our endeavour to attain excellence in OHS, Reliance is investing over Rs.100 crores in the field of OHS.

Our focus is on the key elements of:

1. Process safety
2. Mechanical integrity and quality assurance
3. Capability development
4. Development of corporate safety standards and audit protocols
5. Incident investigation and
6. Contractor safety management

One of the codes of conduct worth highlighting is the chemical industry's initiative, called “Responsible Care”.

This is a public and voluntary commitment by the chemical industry to improve its performance with respect to safety, health and the environment.

Reliance is a signatory to the Responsible Care program and we believe in the good health of the neighbourhood community.

Reliance is committed to improving the quality of life and enhancing the vitality of the communities in which it operates.

Through financial contributions and the voluntary efforts of its employees, Reliance supports programs and organizations that address social progress, economic success and environmental excellence - all vital components of community sustainability.

We have several community health centres at our manufacturing sites. We have adopted nearby villages and render doorstep medical services free of cost.

Various diagnostic and treatment camps, school health check-ups, ante natal clinics, ambulance services, etc. are a regular feature of Reliance.

The successful enterprise in this time of globalization can no longer afford to be a faceless institution that does nothing more than sell the right product at the right price.

But it will have to present itself with a more personalized image, expressing explicit moral judgments when dealing with its own employees, the community and society at large.

Moreover, a product or a workplace that is seen as unsafe by clients or the community, will inevitably affect the image of the enterprise, and will reduce its competitiveness.

We are doing it not only because it the right thing to do, but also because it is our core strategy to grow the company.

## Conclusion

Friends, as is aptly pointed out: Productivity and Profitability go hand in hand with Health and Safety.

To sustain the blistering economic growth and for enhancing quality of life as a whole, OH&S is extremely important to emerging economies.

However, there are many issues that need to be overcome in the areas of awareness, education, training, corporate commitment and regulating compliance.

These challenges can be overcome by employing a combination of appropriate Proactive as well as Reactive strategies.

We at Reliance have immensely benefited by the above mentioned strategies and we remain committed to achieving excellence in this area.

For us, OH&S is not an end in itself but an ongoing journey.

I am sure transitional economies like India will live up to the challenges on OH&S front and aspire for sustainability and economic leadership.

This Ladies and Gentlemen is my fervent hope.

Thank you all for your patience and kind attention.

